# Polyrhythmic foraging and competitive coexistence

**DOI:** 10.1038/s41598-020-77483-3

**Published:** 2020-11-20

**Authors:** Akihiko Mougi

**Affiliations:** Institute of Agricultural and Life Sciences, Academic Assembly, Shimane University, 1060 Nishikawatsu-cho, Matsue, 690-8504 Japan

**Keywords:** Ecology, Ecology

## Abstract

The current ecological understanding still does not fully explain how biodiversity is maintained. One strategy to address this issue is to contrast theoretical prediction with real competitive communities where diverse species share limited resources. I present, in this study, a new competitive coexistence theory-diversity of biological rhythms. I show that diversity in activity cycles plays a key role in coexistence of competing species, using a two predator-one prey system with diel, monthly, and annual cycles for predator foraging. Competitive exclusion always occurs without activity cycles. Activity cycles do, however, allow for coexistence. Furthermore, each activity cycle plays a different role in coexistence, and coupling of activity cycles can synergistically broaden the coexistence region. Thus, with all activity cycles, the coexistence region is maximal. The present results suggest that polyrhythmic changes in biological activity in response to the earth’s rotation and revolution are key to competitive coexistence. Also, temporal niche shifts caused by environmental changes can easily eliminate competitive coexistence.

## Introduction

Diverse species that coexist in an ecological community are supported by fewer shared limited resources than expected, contrary to theory^1^. A simple mathematical theory predicts that, at equilibrium, the number of sympatric species competing for a shared set of limited resources is less than the quantity of resources or prey species^2–4^. This apparent paradox leads ecologists to examine mechanisms that allow competitors to coexist and has produced diverse coexistence theory^5–7^.

Non-equilibrium dynamics is considered as a major driver to prevent interacting species from going into equilibrium and violate the competitive exclusion principle^8,9^. Natural ecosystems, by non-linearity itself, can intrinsically generate non-equilibrium dynamics^10–12^. More fundamentally, externally imposed disturbances, such as seasonal and less predictable changes in environmental conditions, such as weather, also contribute to disequilibrium. Weather shows prominent seasonal cycles. Temperate regions have four obvious seasons, whereas other locations may have fewer or more seasons, and seasons can reflect changes in rainfall as well as temperature and food availability. In any case, our world is undoubtedly seasonal, and species interactions are constrained in a cyclical manner^13–16^.

Earlier ecological theory predicts that temporal variation in environmental conditions and species abundance can create conditions for competitive coexistence^5^. For instance, resource fluctuations or activity cycles of consumers make otherwise impossible coexistence of competing species possible^17–26^. For example, fluctuation plays a role in temporal niche partitioning, which can allow competing species a period of competitive superiority and avoid any one species from being excluded. Yet, previous theories with predictable or deterministic fluctuation have mainly focused on changes occurring within a certain period, such as season^15^; more general environmental fluctuation has multiple periods/cycles.

Generally, organisms are affected by earth cycles, such as earth rotation and revolution^27^. Many organisms have circadian, circalunar, and circannual rhythms, in response to daily, monthly, and annual cycles in environmental conditions. The environment continuously changes over time, and organisms change activities, such as foraging, reproduction, and rest not only within a day but also across longer time scales^28–32^. Some species may sleep during the night, and some species may hibernate during winter. Marine organisms are well known to reproduce on a full or new moon. Besides seasonality, such short-term cycles can affect the strength and structure of species interactions^28–32^. Nevertheless, how the diversity of activity rhythms with different cycle periods affects competitive coexistence remains unclear.

I present in this study a polyrhythmic competition model in which two predators with multiple foraging activity cycles share a single prey. The model considers simple exploitative competition in which two predators with linear functional response compete for a single prey. Population sizes of the superior predator, inferior predator, and prey are represented by *Y*_1_, *Y*_2_, and *X*, respectively. Superior and inferior competitors display higher and lower prey capture rate, *a*_*i*_, respectively (*a*_1_ > *a*_2_). In this system, competitive exclusion always occurs, and coexistence is impossible. I consider multiple periodic cycles of foraging by predators. Daily, monthly, and annual cycles in foraging activity (i.e., capture rate of prey) are described by sine waves with different cyclical periods (1, 30, or 365). The presence or absence of rhythms (daily, monthly, and yearly) is controlled by the amplitudes of the activity cycles, *γ*_d_, *γ*_m_, and *γ*_y_, respectively (*γ*_*i*_ = 1 (*i* = d, m or y) in the presence of a focal rhythm, otherwise 0). In earlier studies, each activity cycle enabled species to coexist if times in activity peaks differed. In the default setting, a perfect difference in activity peaks in predators is assumed. However, depending on the combination of different cycles, the parameter space for coexistence can be greatly broadened. Not all combinations display this result. When all activity cycles are considered, the coexistence region is maximal. The present results suggest that polyrhythmic changes in biological activity are key to maintaining competing species.

## Results

**Figure 1:**
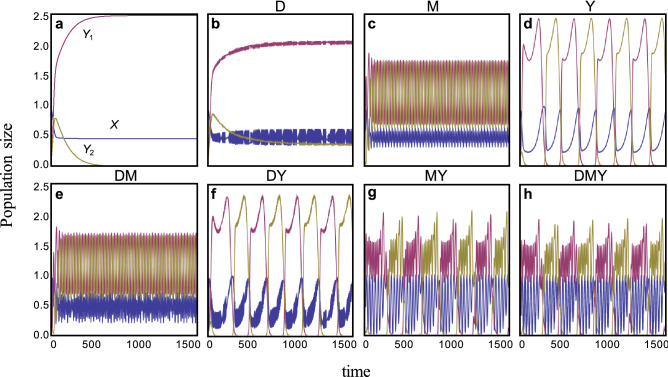
Examples of population dynamics with or without foraging activity cycles. (**a**) No cycles (*γ*_*i*_ = 0). (**b**) Daily cycles (*γ*_d_ = 1, *γ*_m_ = *γ*_y_ = 0). (**c**) Monthly cycles (*γ*_m_ = 1, *γ*_d_ = *γ*_y_ = 0). (**d**) Yearly cycles (*γ*_y_ = 1, *γ*_d_ = *γ*_m_ = 0). (**e**) Daily and monthly cycles (*γ*_d_ = *γ*_m_ = 1, *γ*_y_ = 0). (**f**) Daily and yearly cycles (*γ*_d_ = *γ*_y_ = 1, *γ*_m_ = 0). (**g**) Monthly and yearly cycles (*γ*_m_ = *γ*_y_ = 1, *γ*_d_ = 0). (**h**) All cycles (*γ*_*i*_ = 1). Different colors represent individual species, as shown in the panel (**a**). Parameters are *r* = 5, *a*_01_ = 1.1, *a*_02_ = 1, *g* = 0.2, *d*_*i*_ = 0.1, and *K* = 1.

Without activity cycles (*γ*_*i*_ = 0), competitive exclusion of the inferior species always occurs (Fig. 1a). However, activity cycles allow coexistence. Daily, monthly, and annual cycles can each rescue the inferior predator (Fig. 1b–d). Furthermore, all combinations of coupled cycles also prevent competitive exclusion (Fig. 1e–h).

**Figure 2:**
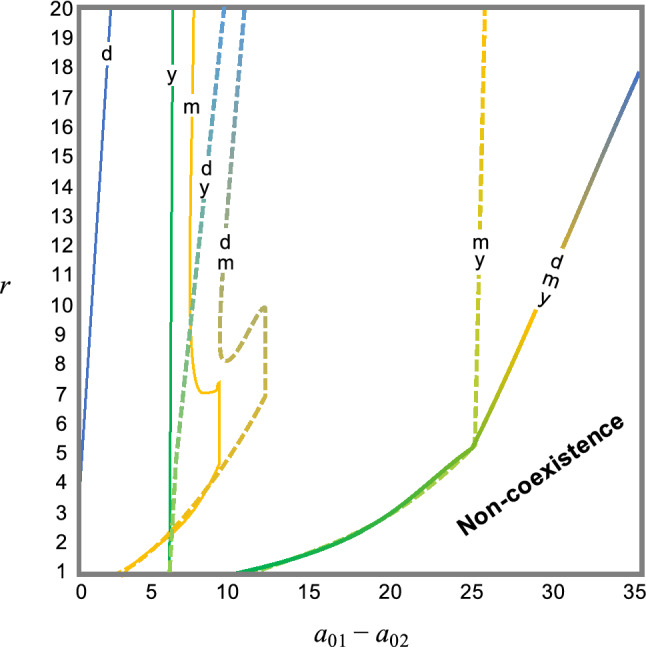
Coexistence regions in the presence of each activity cycle and various combinations of multiple activity cycles. Each line with d, m, y, dy, dm, my, and dmy represents boundaries discriminating coexistence from non-coexistence in the models with daily, monthly, yearly, daily and monthly, monthly and yearly, and all cycles, respectively. In the left side of lines, two predators coexist, and on the other side, competitive exclusion occurs. Parameters are *a*_02_ = 1, *g* = 0.2, *d*_*i*_ = 0.1, and *K* = 1.

The specific combination of activity cycles determines the effect of competition on the coexistence region in a parameter space (Fig. 2). Daily cycles show minimum coexistence regions, and monthly cycles tend to display maximum coexistence regions. Further, coupling cycles expand the coexistence region. The extension of coexistence regions by two cycles is not large, except for one combination. Coupling of monthly and yearly cycles dramatically broadens this region (Fig. 2). Even if the superior predator has a capture rate 25 times the inferior predator, coexistence is possible, depending on prey growth rate.

The coexistence region expands further when all cycles are included. Predators with capture rates that vary by more than a factor of 30 times can coexist (Fig. 2). The rescue effect of activity cycles on coexistence still operates under more severe conditions. Even when the superiority of predators is larger or the inferior predator has a higher death rate, activity cycles permit coexistence (Supplementary Fig. S1).

The above results assume perfect time niche separation. That is, phases of each cycle for the two predators are opposite (for example, one predator is diurnal, and the other is nocturnal). However, phase differences in each activity cycles between predators affect coexistence. When time niches of predators are not perfectly different, coexistence is possible (Supplementary Fig. S2). As time niches overlap, smaller differences between capture rates become necessary. Also, each cycle shows a different response to time niche overlap. Daily and monthly cycles, respectively, have smaller and larger time niche overlap requirements for coexistence (Supplementary Fig. S2).

**Figure 3:**
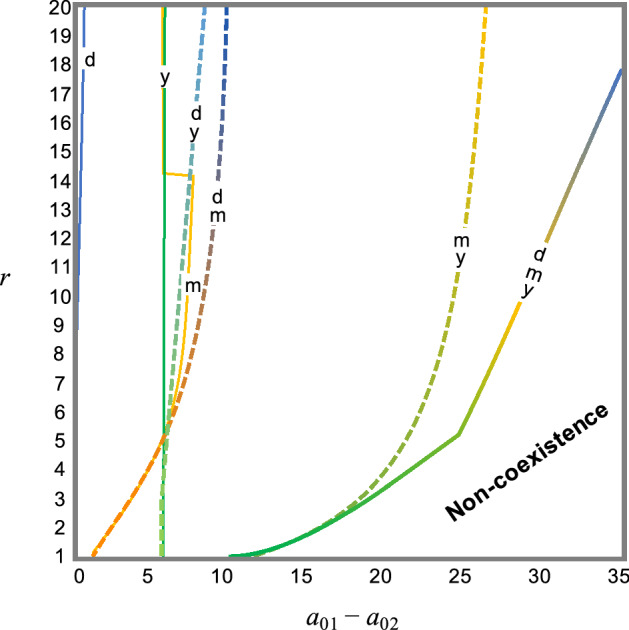
Effects of temporal niche partitioning or overlap on coexistence regions. Predators display all activity cycles (*γ*_*i*_ = 1). In each line represented by d, m, y, dm, dy, my, and dmy, *u*_2d_ = 0.5 (*u*_2m_ = *u*_2y_ = 0); *u*_2m_ = 15 (*u*_2d_ = *u*_2y_ = 0); *u*_2y_ = 365/2 (*u*_2d_ = *u*_2m_ = 0); *u*_2d_ = 0.5 and *u*_2m_ = 15 (*u*_2y_ = 0); *u*_2d_ = 0.5 and *u*_2y_ = 365/2 (*u*_2m_ = 0); *u*_2m_ = 15 and *u*_2y_ = 365/2 (*u*_2d_ = 0); and *u*_2d_ = 0.5, *u*_2m_ = 15, and *u*_2y_ = 365/2, respectively. Parameters are same as parameters in Fig. 2.

I also examined time niche overlap and separation in multiple activity cycles for their effect on coexistence. I assumed that predators display all activity cycles (*γ*_*i*_ = 1). Time niche overlap has a qualitatively similar effect on the coexistence as does lack of any activity cycle (Fig. 3). Niche overlap in daily cycle shows less effect on coexistence. However, overlap in single monthly or annual cycles largely decreases parameter space for coexistence and may show a large negative effect on coexistence comparable with the effect due to overlap in two cycles (Fig. 3). General cases of non-perfect time niche overlap or separation display that increasing time niche overlap gradually decreases the coexistence region (Supplementary Fig. S3), suggesting that a broad parameter space for coexistence can be maintained even if neither temporal niche is perfectly separated.

The present results are valid even if several strong assumptions are relaxed. First, I examined the effects of varying amplitudes of activity cycles (0 < *γ*_*i*_ < 1) (Supplementary Fig. S4). The results demonstrate that coexistence can occur if each activity cycle has an amplitude above a certain level; however, small amplitudes do not allow for coexistence. Additionally, one or two cycles with small amplitudes do not allow for coexistence, but the presence of another cycle with a large amplitude enables coexistence (Supplementary Fig. S4).

For simplicity, the present model does not consider prey activity cycles. Analysis with prey activity cycles shows that a larger difference between the temporal niches of two predators, i.e., temporal niches between prey and one predator are similar, is likely to result in coexistence (Supplementary Fig. S5). Furthermore, although a temporal niche difference in predators of one or two activity cycles is insufficient to enable coexistence, a large temporal niche difference in the other activity cycle allows coexistence (Supplementary Fig. S5). This result also supports the theory that multiple activity cycles prevent competitive exclusion.

To clearly show the importance of multiple activity cycles for species coexistence, a familiar system in which species coexistence is impossible in the absence of external factors was examined in the main analysis. Here, I verify whether the key role of multiple activity cycles for coexistence is valid in other food web modules. I chose two simple and familiar modules, intraguild predation^33^ and apparent competition^34^, in which coexistence is difficult. In the apparent competition system, two prey species that have no direct interaction share a single predator. The indirect competition between prey resulting from shared predation can lead to the extinction of one prey species^34^. In the intraguild predation system, two predators share a single prey species, and one omnivorous generalist predator consumes the other specialist predator. Such a system will collapse if the omnivorous predator outcompetes the specialist predator for the shared prey^33^. I investigated how activity cycles affect these fragile systems in which coexistence is difficult in the absence of activity cycles (Supplementary text). It was revealed that multiple activity cycles allow for otherwise impossible coexistence, thus greatly broadening the coexistence parameter regions in both systems (Supplementary Fig. S6–S8), in a manner similar to the present exploitative competition system.

## Discussion

The present theory predicts that multiple cycles of foraging activity prevent otherwise inevitable competitive exclusion and enables competing species that share a prey species to coexist. Temporal niche partitioning caused by biological cycles allows competitive coexistence, as shown in previous studies^17–26^, and parameter space made by individual cycles allowing coexistence might be much smaller than the space created by multiple cycles. With all activity cycles, coexistence is the least difficult, and, of all pairs of cycles, a combination of monthly and annual cycles creates the greatest space for coexistence. Such combinations show synergistic positive effects. Further, even with niche overlap in any activity cycle, temporal niche differences in other activity cycles still allow for coexistence. Activity cycles play a role in coexistence. Polyrhythms in response to earth cycles may play a key role in maintaining competing species.

Temporal niche partitioning is a classic concept for explaining the coexistence of competing species^28^. In the context of present coexistence theory, a diversity of biological rhythm seems easily understandable, because it assumes multiple temporal niches. However, the effects of individual cycles and, particularly, combinations of cycles on coexistence may not be intuitive. A medium or monthly cycle tends to show a maximal coexistence region, as predicted by the classical intermediate disturbance hypothesis^35^. Daily niche partitioning alone, however, displays a smaller contribution to coexistence, though this effect is not negligible. Everyday reduction of activity of a superior competitor is not enough to suppress the impact of this competitor on an inferior species. Conversely, a long activity cycle or annual niche partitioning alone makes the superior competitor less active during a long time, but it might also imply the opposite situation (more active during a long time). Hence, coexistence may be most favored by an intermediate activity cycle. However, this finding may not be important in nature since organisms should show multiple activity cycles.

The present theory suggests that multiple activity cycles have complementary and synergistic effects for species coexistence. Weak interaction is a key factor in coexistence. As demonstrated by the effects of cycle amplitude (Supplementary Fig. S4), variation in activity is necessary for coexistence. This suggests that the “rest” periods of activity cycles play a key role in the coexistence mechanism. In fact, multiple activity cycles produce greater variation in activity levels (Supplementary Fig. S9). More importantly, the hybridization of activity cycles contributes to a lower activity level distribution (Supplementary Fig. S10); when all cycles are mixed, the skew toward low activity reaches a maximum (Supplementary Fig. S10). These results suggest that, combined with temporal niche partitioning, temporal low activity or “rest” periods in multiple activity cycles can greatly reduce species interaction, which is essential for maintaining competing species.

The model has several important biological implications. First, a daily time niche may not be less important for coexistence than monthly or annual niches. This conclusion is supported by empirical observation. For example, daily activity time of large carnivores competing for limited resources in the African guild is highly overlapped, but their activities caused by the lunar cycle are clearly different^36^. Conversely, multiple activity cycles may support species coexistence. Ecologically similar lemurs in the lowland rainforest of Tsitongambarika in south-eastern Madagascar show temporal niche partitioning by day and month^37^. Sympatric ocelots and bobcats in South Texas use different daily, monthly, and annual niches^38^. Other partial evidence may come from fish communities. A distinct seasonal pattern shown in abundances of fish groups coexisting in the Bristol Channel can be driven by monthly effects^39^. The mechanisms underlying this pattern are difficult to differentiate, but the monthly and yearly temporal variations most likely contribute to species coexistence. These observations do not provide direct evidence of the theoretical prediction; therefore, laboratory experiments and appropriate observational data are required to fully test the theory. This can be tested by comparing the coexistence times among systems with competing species with different and similar rhythms. If the different rhythms prolong the coexistence time, indirect evidence of the theory will be obtained. Another approach to obtain indirect evidence is through natural observations of the time niche differences between closely-related species that locally coexist in one place and allopatrically in different places. It is expected that the locally-coexisting competing species will exhibit larger time niche differences and/or niche differences in multiple time niches compared with the allopatrically coexisting competing species. In addition, comparing competing species with similar time niches will highlight the differences in other resource niches among them, like diet and habitat. Elucidating the relationships between different types of niches through detailed observation of the temporal niches of competing species would help to test the theory. For example, coexisting species with a greater niche overlap in their diet may have larger temporal niche differences and/or differences in multiple temporal niches, and vice versa.

Finally, the synergistic effect of multiple activity cycles on coexistence has major implications for biological conservation. Environmental influences such as artificial light at night^40^, temperature rise^32,41^, and climatological changes^42^, can alter activity levels, patterns, or periods in daily, monthly, and annual cycles, respectively. If any temporal niche, particularly a monthly or annual niche, overlaps among competing species, coexistence can be destroyed by concomitant shrinking of the coexistence regions. Furthermore, if different activity rhythms are interrelated^43^, disorder in one activity cycle can disturb another. Thus, simultaneous disruption of multiple activity rhythms can also impact coexistence. Tracing the activity patterns and population dynamics of competing species that share limited resources is essential for testing this theory and subsequently forming conservation strategies. The present study also provides a foundation to further develop community models with multiple activity cycles. Adaptive temporal niche shifts through phenotypic plasticity or evolution^44,45^, which can change patterns of multiple activity cycles, are a key element for the application of the present theory to a more general one.

## Methods

Consider two competing predator species that share a single prey species. The simplest two predator–one prey system is defined by the following ordinary differential equations:

1 $$ \frac{dX}{{dt}} = r\left( {1 - \frac{X}{K}} \right)X - a_{1} XY_{1} - a_{2} XY_{2} , $$

2 $$ \frac{{dY_{1} }}{dt} = g_{1} a_{1} XY_{1} - d_{1} Y_{1} , $$

3 $$ \frac{{dY_{2} }}{dt} = g_{2} a_{2} XY_{2} - d_{2} Y_{2} , $$

where *X* is the abundance of prey species; *r* is the intrinsic rate of change in prey species; *K* is the carrying capacity of prey species; *a*_*i*_ (*i* = 1 or 2) is capture rate (i.e., the rate at which the predator captures its prey); *g*_*i*_ is conversion efficiency, which relates the predator’s birth rate to prey consumption (*g*_*i*_ = *g* is assumed for simplicity); and *d*_*i*_ is the death rate of predator species *i*.

Here, *a*_*i*_ = *a*_0*i*_c_*i*_(t), where *a*_0*i*_ is the basal capture rate. c_*i*_(t) is a time-varying function, which represents daily, monthly, annual cycles of foraging activity, or polyrhythmic cycles made by combinations of cycles. Each basal biological cycle is described by the following sinusoidal functions^24^:

4 $$ {\text{c}}_{i} \left( {\text{t}} \right) \, = { 1 } + {\text{ sin}}\left\{ {{2}\pi \left( {{\text{t }}{-}u_{i} } \right)/T_{j} } \right\}, $$

where *T*_*j*_ and *u*_*ij*_ are cycle period and timing of activity peak (*j* = *d*, *m*, or *y*, each representing day, month, and year). Here, *T*_*d*_ = 1, *T*_*m*_ = 30, and *T*_*y*_ = 365. For the daily cycle, *u*_*id*_ may be 0 or 1/2, defined as diurnal or nocturnal, respectively. For the monthly cycle, *u*_*im*_ may be 0 or 30/2, each defined as full moon or new moon type, respectively. For the yearly cycle, *u*_*iy*_ may be 0 or 365/2, each defined as summer or winter type, respectively. In this study, *u*_1*j*_ = 0, and the default values of *u*_2*d*_, *u*_2*m*_, and *u*_2*y*_ are 0.5, 15, and 365/2, respectively. This restrictive assumption is later relaxed).

Models with either two or all cycles are given by the product of each cycle function with different cycle periods.

5 $$ {\text{c}}_{i} \left( {\text{t}} \right) = \prod\limits_{j} {\left[ {1 + \gamma_{j} \sin \left\{ {2\pi \left( {t \, {-}u_{ij} } \right)/T_{j} } \right\}} \right]} , $$

where *γ*_*j*_ is the parameter that controls presence (*γ*_*j*_ = 1) or absence (*γ*_*j*_ = 0) of cycles.

For example, in the case with daily and monthly cycles (*γ*_*d*_ = 1, *γ*_*m*_ = 1, *γ*_*y*_ = 0), c_*i*_(t) = [1 + sin{2π(t − *u*_*id*_)/*T*_*d*_}][1 + sin{2π(t − *u*_*im*_)/*T*_*m*_}], and in the case with all cycles, c_*i*_(t) = [1 + sin{2π(t − *u*_*id*_)/*T*_*d*_}][1 + sin{2π(t − *u*_*im*_)/*T*_*m*_}][1 + sin{2π(t − *u*_*iy*_)/*T*_*y*_}]. Single cycles are assessed with Eq. (4). Note that each function with multiple periods has the same mean value, 1. Using this parameter allows appropriate comparisons with each model, including the null model without biological cycle.

Without activity cycles (*γ*_*j*_ = 0), c_*i*_(t) = 1, the system is a well-known Lotka–Volterra type two predator–one prey system. This system produces no coexistence equilibrium. That is, one competing species is always competitively excluded. The system converges to one of the following equilibria: X^*^ = *d*_1_/*a*_1_, *Y*_1_^*^ = *r*(*a*_1_ − *d*_1_/*K*)/*a*_1_^2^, *Y*_2_^*^ = 0 or X^*^ = *d*_2_/*a*_2_, *Y*_1_^*^ = 0, *Y*_2_^*^ = *r*(*a*_2_ − *d*_2_/*K*)/*a*_2_^2^.

Models with activity cycles are analytically intractable. To examine the population dynamics of three species and evaluate competitive coexistence, I performed a numerical simulation of the differential Eqs. (1)–(3) by using a numerical technique NDSolve in Mathematica. The initial values of each species were set to 0.1. If competing species co-occurred (*Y*_*i*_ > 10^−4^ for all *i*) after sufficient time (t) t = 5 × 10^3^, which corresponds with the time taken for community persistence to reach an asymptote, it is evaluated as competitive coexistence (otherwise competitive exclusion).

## Supplementary information

Supplementary Information.

## Data Availability

All data generated and analysed during this study are included in this published article.
