# Supplementary material for: Polyrhythmic foraging and competitive coexistence
Source: Sci Rep. 2020 Nov 20;10:20282. doi: 10.1038/s41598-020-77483-3 (PMC7679447; doi:10.1038/s41598-020-77483-3)

Supplementary Information

Polyrhythmic foraging and competitive coexistence

A. Mougi

Several relaxations of the model and some extension to other food web modules were tested in this supplement. The effects of asymmetry of death rates, varying time niche difference and varying amplitudes of activity cycles are shown in Figures S1, S2 and S3, and S4, respectively. Figure S5 shows a case where prey also has activity cycles. Figures S6 and S7 show a case where one predator with activity cycles consumes two preys. Figure S8 is an intraguild predation system where one predator consumes both one prey and another predator. In Figs S9 and S10, the dynamics and distribution of activity levels are shown, which helps to understand the coexistence mechanism due to multiple activity rhythms. The hybridity of multiple rhythms causes activity cycles with large amplitude (Fig S9) and skews the activity levels toward weak (Fig S10). The weak interaction is considered to be a key mechanism for species coexistence.

Prey's activity cycles are modelled as: $r = r_0 \prod_j \left[1+\gamma_j \sin\left(2\pi t / T_j\right)\right]$, where r_0 is the basal growth rate in prey species.

One predator-two prey system is modelled as below:

$$\frac{dX_1}{dt} = r_1\left(1-\frac{X_1}{K_1}\right)X_1 - a_1X_1Y, \qquad \text{(S1a)}$$

$$\frac{dX_2}{dt} = r_2\left(1-\frac{X_2}{K_2}\right)X_2 - a_2X_2Y, \qquad \text{(S1b)}$$

$$\frac{dY}{dt} = g\left(a_1X_1 + a_2X_2\right)Y - dY, \qquad \text{(S1c)}$$

where X_i is the abundance of each prey species; r_i is the intrinsic rate of change in each prey species; K_i is the carrying capacity of each prey species; a_i (i = 1 or 2) is capture rate (i.e., the rate at which the predator captures each prey species); g is conversion efficiency, which relates the

predator's birth rate to prey consumption; and d is the death rate of predator species. Here, $a_i = a_{0i} \prod_j \left[1+\gamma_j \sin\left(2\pi t / T_j\right)\right]$, where a_{0i} is the basal capture rate of each prey species.

Here, consider a case without activity cycles ($\gamma_i = 0$). For the analytical simplicity, two preys have same parameters except for interaction coefficients ($r_i = r$ and $K_i = K$). The basic model has a coexistence equilibrium: $X_1^* = \dfrac{a_1 d - a_2(a_1 - a_2)gK}{(a_1^2 + a_2^2)g}$, $X_2^* = \dfrac{a_2 d + a_1(a_1 - a_2)gK}{(a_1^2 + a_2^2)g}$ and $Y^* = \dfrac{r\{(a_1 + a_2)gK - d\}}{(a_1^2 + a_2^2)gK}$. The feasible condition ($X_1^*, X_2^*, Y^* > 0$) is:

$$\frac{d}{gK} > a_2\left(1 - \frac{a_2}{a_1}\right), \tag{S2a}$$

$$\frac{d}{gK} > a_1\left(1 - \frac{a_1}{a_2}\right), \tag{S2b}$$

$$\frac{d}{gK} < a_1 + a_2, \tag{S2c}$$

In the analysis, a prey species 1 has higher defensive ability than prey 2 ($a_1 < a_2$). Hence, the condition (S2a) is always true. Then, the feasible condition is reduced to:

$$a_1 + a_2 > \frac{d}{gK} > a_1\left(1 - \frac{a_1}{a_2}\right) \tag{S3}$$

As shown in Fig. S6 and S7, activity cycles allow coexistence even when the last condition is not met.

Intraguild predation system is the following:

$$\frac{dX}{dt} = r\left(1 - \frac{X}{K}\right)X - a_1 X Y_1 - a_2 X Y_2, \quad \text{(S4a)}$$

$$\frac{dY_1}{dt} = g\left(a_1 X + a_3 Y_2\right)Y_1 - d_1 Y_1, \quad \text{(S4b)}$$

$$\frac{dY_2}{dt} = g_2 a_2 X Y_2 - a_3 Y_2 Y_1 - d_2 Y_2, \quad \text{(S4c)}$$

where a_3 is the capture rate of predator species 1 for predator species 2. $a_3 = a_{03} \prod_j \left[1 + \gamma_j \sin\left(2\pi t / T_j\right)\right]$, where a_{03} is the basal capture rate of predator species 1 for predator species 2. The other parameters and notations are same with those of the model in the main text.

Here, consider a case without activity cycles ($\gamma_i = 0$). For the analytical simplicity, $d_i = d$. Here, consider a situation where predator species 2 invades into a community comprised of prey and predator 1. The coexistence equilibrium is: $X^* = d/ga_1$ and $Y_1^* = r(a_1 gK - d)/a_1^2 gK$. Under the equilibrium, the invasion of predator 2 is possible if dY/dt$Y$ > 0. The invasion condition is the following:

$$a_1 a_3 Y_1^*/d < - a_1 + a_2 \quad \text{(S5)}$$

Because $a_1 > a_2$ in the present analysis, it is clear that the invasion condition (S5) is always not met. Under this severe situation for predator 2, however, activity cycles allow the coexistence of predators easily, as shown in Fig. S8.

Supplemental Figures

S1 Fig. Effects of asymmetry of death rates on coexistence regions. Each line represents boundaries discriminating coexistence or non-coexistence in the models with different individual activity cycles or different combinations of activity cycles, as shown in Fig. 2. Parameters are $r = 15$, $a_{02} = 1$, $g = 0.2$, $d_2 = 0.1$, and $K = 1$.

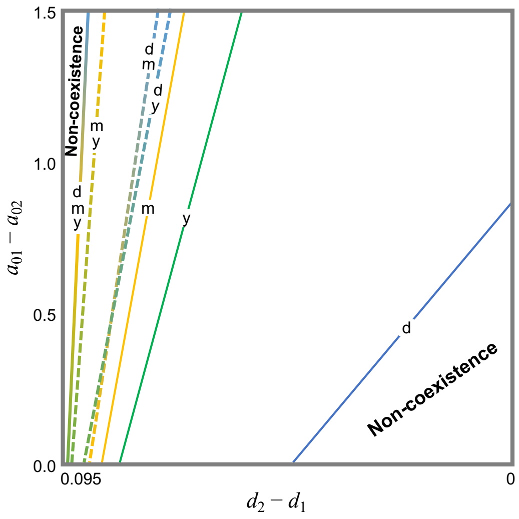

S2 Fig. Effects of time niche difference on coexistence regions. Each line represents boundaries discriminating coexistence and non-coexistence in models with daily, monthly, and yearly cycles. The horizontal axes are normalized time niche differences, defined as $\left|u_{2j} - u_{1j}\right| / \hat{u}_{2j}$, where $\hat{u}_{2j}$ is the maximum value of timing of activity peak in each cycle ($\hat{u}_{2d} = 0.5$, $\hat{u}_{2m} = 15$, and $\hat{u}_{2y} = 365/2$). Parameters are $r = 15$, $a_{02} = 1$, $g = 0.2$, $d_i = 0.1$, and $K = 1$.

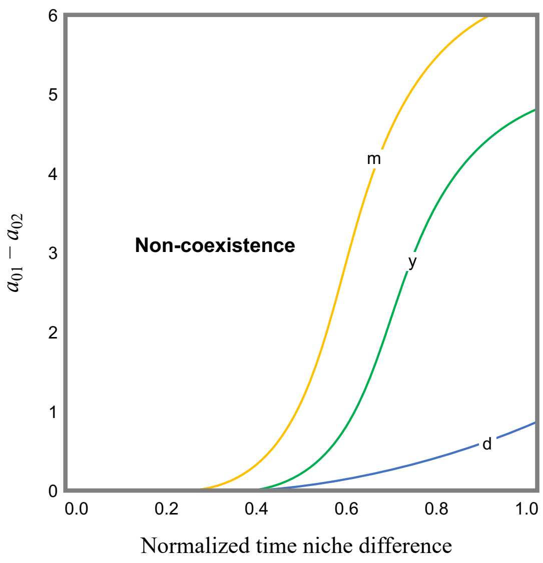

S3 Fig. Effects of temporal niche differences on coexistence. (a) Effects of annual niche difference. (b) Effects of monthly niche difference. Predators display all activity cycles ($\gamma_i = 1$). Different lines in each panel represent differences from perfect niche separation, depicted by solid color lines where $u_{2y} = 365/2$ in (a) and 15 in (b). Dotted lines represent cases of perfect niche overlap. Parameters are the same as parameters in Fig. 2.

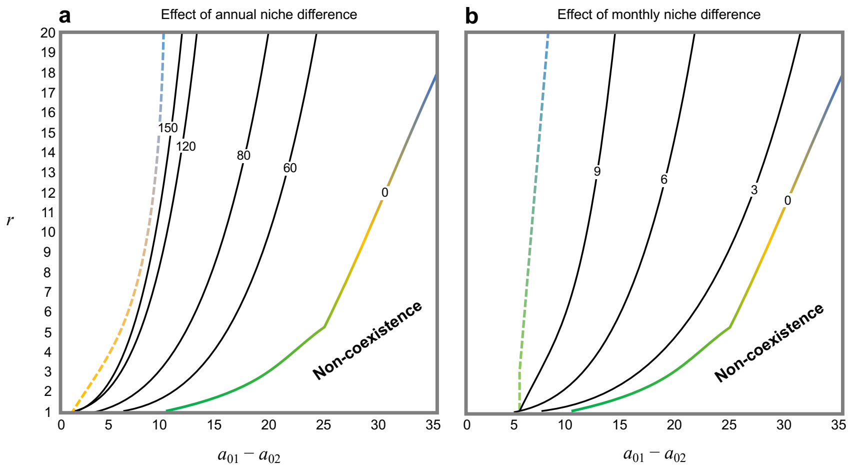

S4 Fig. Effects of amplitudes of activity cycles on coexistence. Each line with different colors represents boundaries between coexistence and non-coexistence regions in cases with different values of γ_{d}. Parameters are $r = 5$, $a_{01} = 3$, $a_{02} = 1$, $g = 0.2$, $d_i = 0.1$, and $K = 3$.

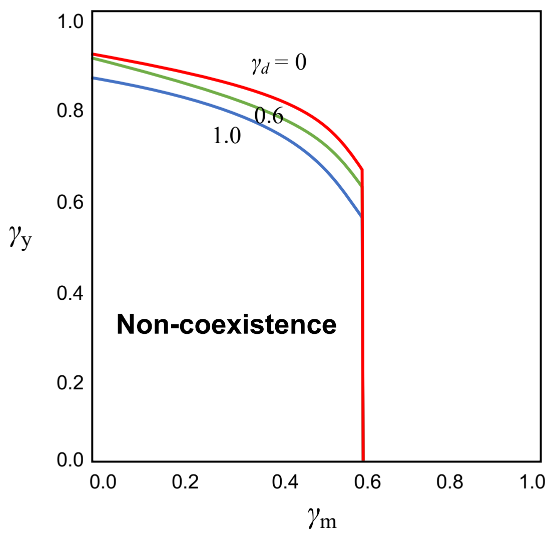

S5 Fig. Effects of time niche difference between predators on coexistence in the model with prey activity cycles. δ_d (= $u_{1d} - u_{2d}$), δ_m (= $u_{1m} - u_{2m}$) and δ_y (= $u_{1y} - u_{2y}$) represents time niche difference between predators in day, month and year, respectively. Parameters are u_{1d} = 0.5, u_{1m} = 15, u_{1y} = 365/2, γ_i = 1, r = 5, a_{01} = 5, a_{02} = 1, g = 0.2, d_i = 0.1, and K = 3.

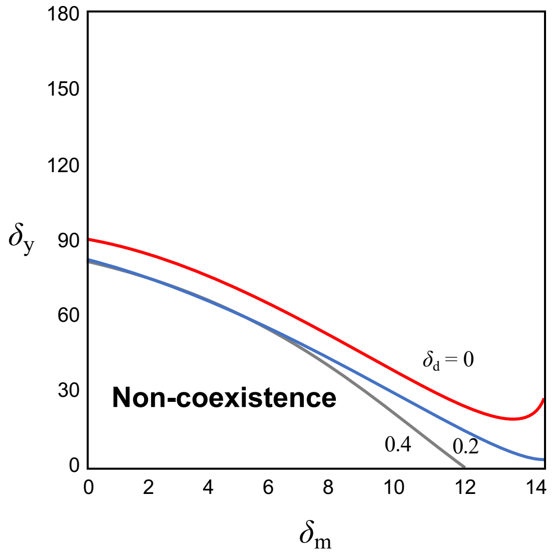

S6 Fig. Effects of activity cycles on coexistence in one predator-two prey system. (a) Without activity cycles (γ_i = 0). (b) With activity cycles (γ_i = 1). Each dotted line with different colors represent mean population size over 1000 time steps after sufficient time (t) t = 5 × 10^3, which corresponds with the time taken for community persistence to reach an asymptote. Black arrows represent coexistence region. Networks shown at the bottom of the panels represent food web modules corresponding to each value of a_{02}. Parameters are r_i = 1, a_{01} = 1, g = 0.2, d_i = 0.1, and K_i = 1.

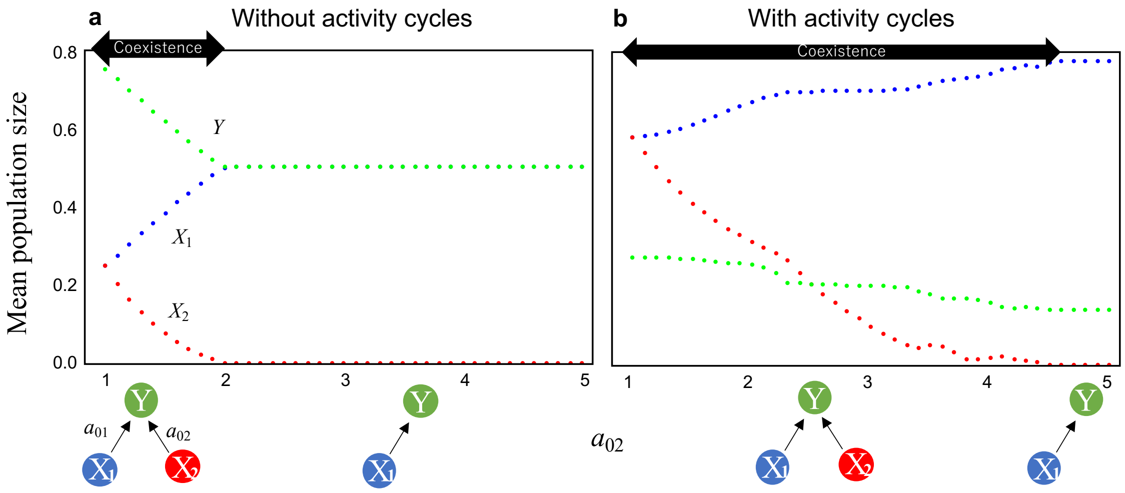

S7 Fig. Examples of population dynamics with or without foraging activity cycles in one predator-two prey system. (a) No cycles ($\gamma_i = 0$). (b) Daily cycles ($\gamma_d = 1, \gamma_m = \gamma_y = 0$). (c) Monthly cycles ($\gamma_m = 1, \gamma_d = \gamma_y = 0$). (d) Yearly cycles ($\gamma_y = 1, \gamma_d = \gamma_m = 0$). (e) Daily and monthly cycles ($\gamma_d = \gamma_m = 1, \gamma_y = 0$). (f) Daily and yearly cycles ($\gamma_d = \gamma_y = 1, \gamma_m = 0$). (g) Monthly and yearly cycles ($\gamma_m = \gamma_y = 1, \gamma_d = 0$). (h) All cycles ($\gamma_i = 1$). Different colors represent individual species, as shown in the panel (a). Parameters are $r_i = 1$, $a_{01} = 1$, $a_{02} = 4$, $g = 0.2$, $d_i = 0.1$, and $K_i = 1$.

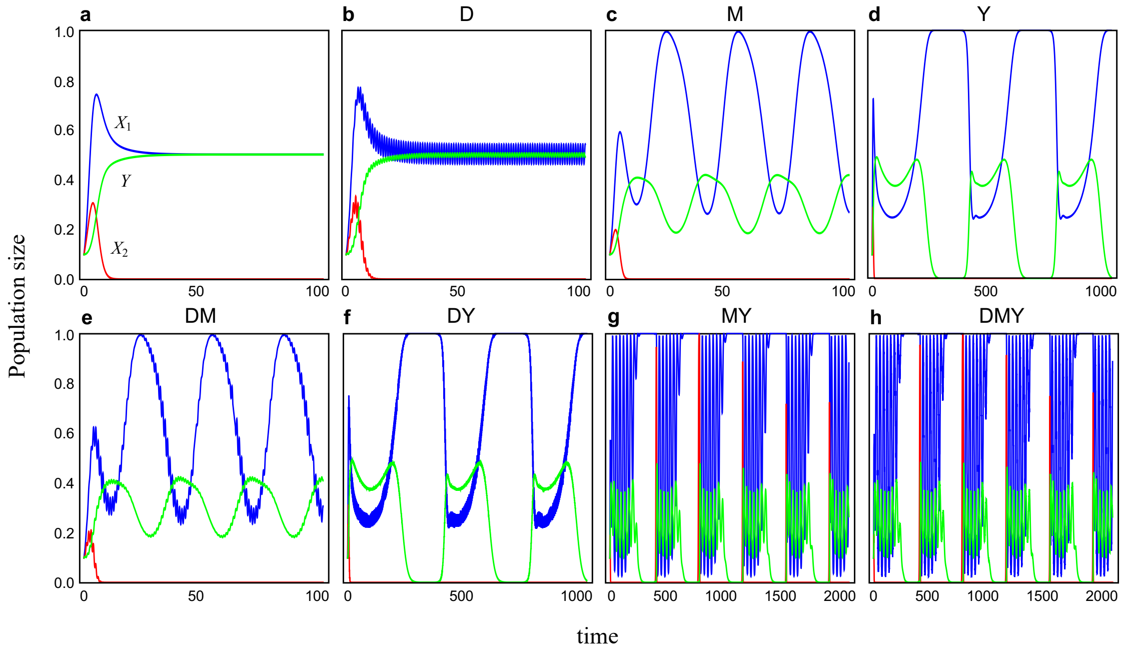

S8 Fig. Effects of strength of intraguild predation on coexistence in model with intra-guild predator. Different dotted lines represent mean population size of each species over 1000 time steps after sufficient time (t) t = 5 × 10^3, which corresponds with the time taken for community persistence to reach an asymptote. Black arrows represent coexistence region. Networks shown at the bottom of the panels represent food web modules corresponding to each value of a_{02}. Parameters are $\gamma_i = 1$, $r = 1$, $a_{01} = 5$, $a_{02} = 1$, $g = 0.2$, $d_i = 0.1$, and $K_i = 3$.

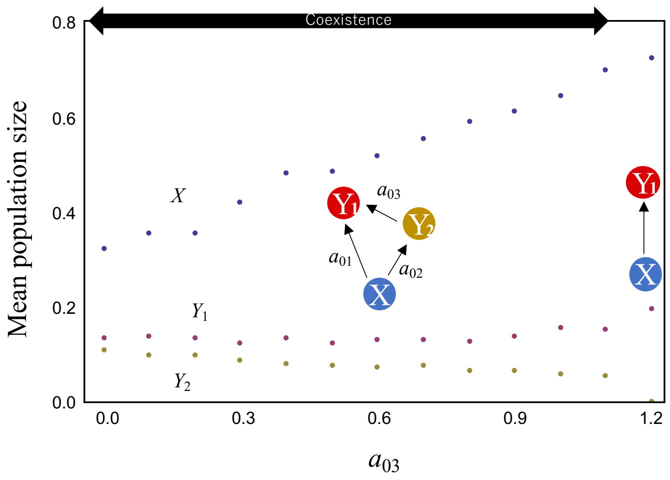

S9 Fig. Diversity of activity cycles, $c_i(t)$. (a) Daily and Monthly cycles. (b) Daily and Yearly cycles. (c) Monthly and Yearly cycles. (d) All cycles. In (a-c), each single cycle is shown by red and blue lines. Standard Deviation (sd) of each activity level is: 0.71 (single cycle), 1.12 (two cycles) and 1.55 (all cycles).

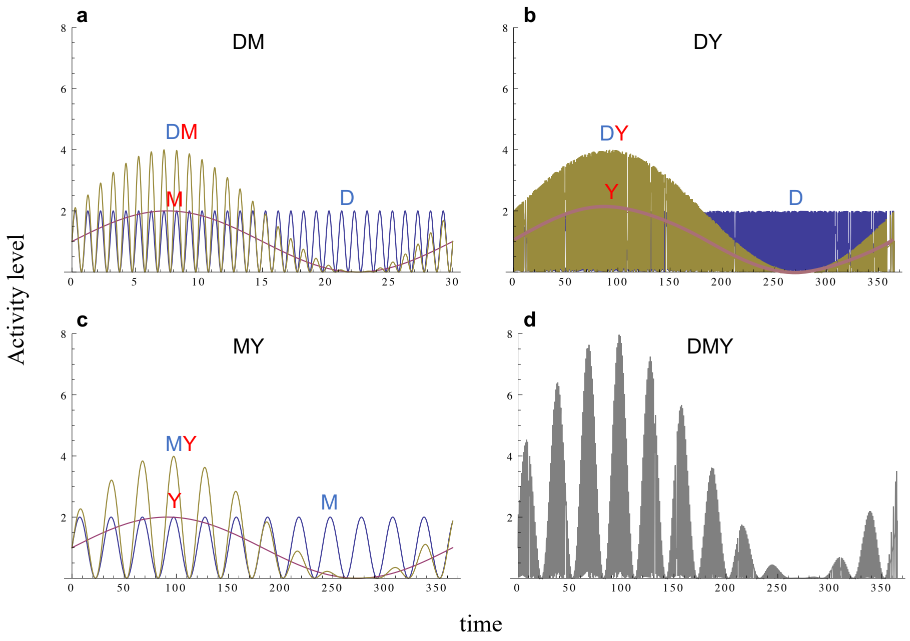

S10 Fig. Histgram of activity levels in each activity cycle. (a) Single cycle. Blue, red and yellow represents daily (D), monthly (M) and yearly (Y) cycle, respectively. (b) Two cycles. Blue, red and yellow represents DM, DY and MY, respectively. (c) All cycles. (d) Comparison between MY and DMY. The number of data points are $4{\times}10^5$.

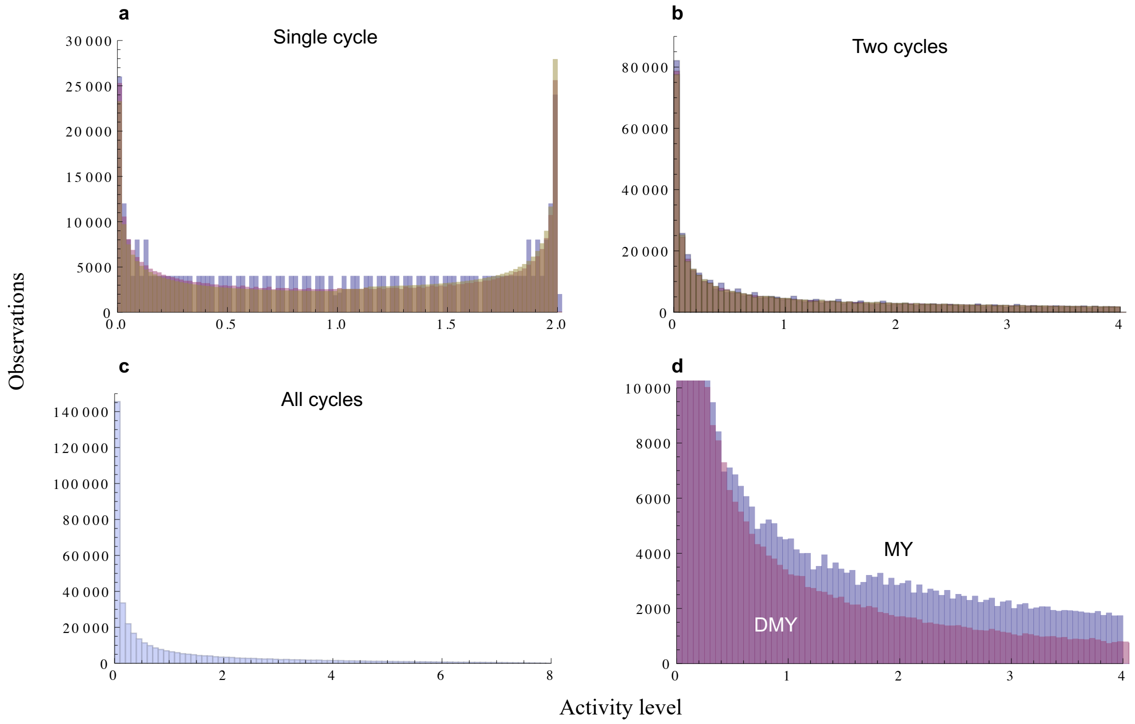

Supplement: Supplementary file 1 — Supplementary Information. [file 41598_2020_77483_MOESM1_ESM.pdf]
